# Nitrous Oxide as an Anæsthetic

**Published:** 1864-05

**Authors:** Geo. J. Ziegler


Nitrous Oxide as an Anæsthetic.—*By Geo. J. Ziegler, M. D.*—As much misapprehension appears to exist respecting the physiological influences of protoxide of nitrogen, which is again in favor for anæsthetic purposes, a few general remarks upon its constitution, medical properties, and correlations may not be devoid of practical value.

In the first place, then, with regard to the predominant characteristics of nitrous oxide it is truly *sui generis*, though closely allied in chemical constitution, material properties, and sanative effects with atmospheric air, which may be regarded, in fact, as its natural prototype, differing therefrom apparently more in the proportion of its constituent elements—nitrogen and oxygen—and in the manner of their association, than in any other essential respect, although its action upon the animal economy is more intense, concentrated, and manifest than that of the latter, varying rather in the degree, perhaps, than in the nature of its physiological influence. While, however, there is thus a general correlation between nitrous oxide and atmospheric air, yet each one has special peculiarities of its own, of such a marked character, indeed, as to make them appear quite distinct and render them useful for diverse as well as similar purposes.

The effects of protoxide of nitrogen upon the human system vary in proportion to the quantity appropriated and the peculiar susceptibilities of individual organisms, passing from a gentle acceleration of all the functions of the body to a high degree of physical excitement and mental exhilaration, even to a sort of delirium or extasy of a highly pleasurable character, during the existence of which the mind is apparently unconscious of, or indifferent to, impressions of an ordinarily painful nature. This delirium is usually, however, of brief duration, terminating somewhat suddenly, yet leaving generally a sense of permanent invigoration similar to that resulting from a free exposure to fresh atmospheric air. Besides its general physiological effects, nitrous oxide has a special tendency to the blood, brain, nervous system, and genito-urinary organs.

By thus intensifying the general functions of life, protoxide of nitrogen produces such a high degree of exhilaration as to render the mind temporarily unconscious of, or indifferent to, impressions which would otherwise occasion pain and suffering. Notwithstanding this condition of mind and body is quite distinct from that caused by ether, chloroform, and similar agents, yet it is attended in some measure with the same general results in the production of a state of insensibility, though not of stupefaction, sufficient in degree to admit of certain surgical manipulations without the usual concomitant—pain.

In chemical constitution and properties, as well as in its physiological influences, nitrous oxide differs from all other anæsthetics, for these latter are not only chemically dissimilar, but are always, more or less directly sedative in their action upon the animal organism; whereas the former is *ab initio* primarily and permanently stimulant, not even being followed with any of that languor and depression so peculiar to the others, yet while not altogether devoid of danger, it is still much less in degree, and mostly of an entirely different character from those referred to.

Instead therefore of resembling, nitrous oxide differs essentially both in constitution and properties from the ordinary anæsthetics, and is, in fact, directly antagonistic and antidotal thereto, its anæsthetic effect being the result of a stimulant, and not of a sedative action.

Protoxide of nitrogen is thus not only superior in chemical constitution and the nature of its primary effects upon the economy, but also, in leaving a permanent feeling of general invigoration instead of that sedation always in a greater or less degree attending the action of ordinary anæsthetics; and, moreover, in being in a large measure, unattended with that immediate or subsequent danger which render the latter, in the main, so objectionable.

While, however, the physiological effects of nitrous oxide are usually of a highly pleasurable and sanative character, it can not, nevertheless, be indiscriminately employed with safety, for the artificial excitement of system rapidly engendered by its free administration may not only prove injurious by directly increasing the tendency to irritation, hæmorrhage, and inflammation in the parts subjected to surgical mutilation, but may also develop latent pathological tendencies of a different as well as of a like character in other parts of the body, in persons with certain abnormal predispositions, to such a degree, indeed, as to seriously injure health, if not absolutely endanger life itself.

The precise character and particular manifestation of such tendencies will, of course, depend upon the special predisposition of the individual system acted upon, but they will necessarily be most likely to appear in certain definite part, of the body in accordance with the peculiarities of action of the disturbing agent—nitrous oxide having, as before stated, a marked preference for the blood, brain, nervous systems and genito-urinary organs.

These brief observations will suffice to show the general character of the dangers to be apprehended from the undue or injudicious administration of nitrous oxide, yet as they may not be sufficiently definite for practical purposes, I will present a more detailed notice of these extraneous tendencies.

Thus, for instance, the undue excitement occasioned by the free or inappropriate use of protoxide of nitrogen may produce both primary and secondary irritation, congestion, serous or hæmorrhagic effusion, and inflammation in different parts of the body, and especially in the brain and kidneys. Besides, principally by superoxidation and over-stimulation, it may cause excessive disintegration and undue waste as well as abnormal excitement of the system, even to destructive softening of the brain, nervous tissue, and other important structures. Furthermore, by unduly accelerating functional action it may give rise to rupture of the heart and blood-vessels, or disruption and other mechanical derangements of important parts of the organism. Moreover, through its powerful aphrodisiac effects it may intensify sexual desire to such a degree as to cause unpleasant exposure or even serious trouble.

In view, therefore, of the highly important considerations for protecting the general health and insuring the safety of those subjected to its influence, as well as for the morality of the patient and reputation of the operator, nitrous oxide should always be administered with great care and precaution.

Notwithstanding, however, these apparently serious objections to the free use of protoxide of nitrogen would seem to strongly militate against its general employment for anæsthetic and other purposes, yet they are materially diminished in force by the compensating fact, that it has, in some measure, the ability to counteract such tendencies, whether antecedent or consequent, through its material and dynamic power to aerate, depurate, and increase the plasticity of the blood, encourage healing by first intention, regulate innervation, circulation, nutrition, contractability and other essential functions; and, by its general systemic invigoration and highly sanative influence, to protect the living organism against any temporary or permanent injury. While in this way some of the dangerous tendencies of this agent are counteracted, it is, nevertheless, always necessary to bear in mind that such do exist and can not even be slightly disregarded without peril, for evil may follow when least expected; hence an enlightened judgment and a judicious discrimination are constant prerequisites in the administration of nitrous oxide, in order to form a correct estimate of contra-indicating circumstances, and guard against injurious results.

These precautionary remarks are not made with any disposition to undervalue this remarkable agent or excite undue apprehension respecting the potency of its action upon the economy, but simply with a view to afford a correct exposition of its anæsthetic and other properties, for no one has a higher appreciation of its intrinsic merits and valuable sanative effects than myself, yet it is proper that the truth should be known, to enable all to avoid the evil and obtain the good.

The general sanative properties and applications of protoxide of nitrogen are numerous, diversified, and important, but as I have before treated of these somewhat at length and hope soon again to present elsewhere some general observations thereupon, I will not invite further attention to them in this place, as they are not so immediately pertinent to the practice of dentistry.

